# Preoperative Heart Rate Variability During Sleep Predicts Vagus Nerve Stimulation Outcome Better in Patients With Drug-Resistant Epilepsy

**DOI:** 10.3389/fneur.2021.691328

**Published:** 2021-07-07

**Authors:** Xi Fang, Hong-Yun Liu, Zhi-Yan Wang, Zhao Yang, Tung-Yang Cheng, Chun-Hua Hu, Hong-Wei Hao, Fan-Gang Meng, Yu-Guang Guan, Yan-Shan Ma, Shu-Li Liang, Jiu-Luan Lin, Ming-Ming Zhao, Lu-Ming Li

**Affiliations:** ^1^National Engineering Laboratory for Neuromodulation, School of Aerospace Engineering, Tsinghua University, Beijing, China; ^2^Medical Innovation Research Division, Research Center for Biomedical Engineering, Chinese People's Liberation Army General Hospital, Beijing, China; ^3^Department of Neurosurgery, Beijing Neurosurgical Institute, Beijing, China; ^4^Department of Neurosurgery, Beijing Tian Tan Hospital, Capital Medical University, Beijing, China; ^5^Department of Neurosurgery, Sanbo Brain Hospital Capital Medical University, Beijing, China; ^6^Department of Neurosurgery, Peking University First Hospital FengTai Hospital, Beijing, China; ^7^Department of Neurosurgery, Beijing Children's Hospital, Capital Medical University, Beijing, China; ^8^Department of Neurosurgery, Tsinghua University Yuquan Hospital, Beijing, China; ^9^Department of Neurosurgery, Aerospace Center Hospital, Beijing, China; ^10^Precision Medicine and Healthcare Research Center, Tsinghua-Berkeley Shenzhen Institute, Shenzhen, China; ^11^Institute of Human-Machine, School of Aerospace Engineering, Tsinghua University, Beijing, China; ^12^Center of Epilepsy, Beijing Institute for Brain Disorders, Beijing, China

**Keywords:** drug-resistant epilepsy, heart-rate variability, vagus nerve stimulation, circadian rhythm, outcome prediction, feature selection

## Abstract

**Objective:** Vagus nerve stimulation (VNS) is an adjunctive and well-established treatment for patients with drug-resistant epilepsy (DRE). However, it is still difficult to identify patients who may benefit from VNS surgery. Our study aims to propose a VNS outcome prediction model based on machine learning with multidimensional preoperative heart rate variability (HRV) indices.

**Methods:** The preoperative electrocardiography (ECG) of 59 patients with DRE and of 50 healthy controls were analyzed. Responders were defined as having at least 50% average monthly seizure frequency reduction at 1-year follow-up. Time domain, frequency domain, and non-linear indices of HRV were compared between 30 responders and 29 non-responders in awake and sleep states, respectively. For feature selection, univariate filter and recursive feature elimination (RFE) algorithms were performed to assess the importance of different HRV indices to VNS outcome prediction and improve the classification performance. Random forest (RF) was used to train the classifier, and leave-one-out (LOO) cross-validation was performed to evaluate the prediction model.

**Results:** Among 52 HRV indices, 49 showed significant differences between DRE patients and healthy controls. In sleep state, 35 HRV indices of responders were significantly higher than those of non-responders, while 16 of them showed the same differences in awake state. Low-frequency power (LF) ranked first in the importance ranking results by univariate filter and RFE methods, respectively. With HRV indices in sleep state, our model achieved 74.6% accuracy, 80% precision, 70.6% recall, and 75% F1 for VNS outcome prediction, which was better than the optimal performance in awake state (65.3% accuracy, 66.4% precision, 70.5% recall, and 68.4% F1).

**Significance:** With the ECG during sleep state and machine learning techniques, the statistical model based on preoperative HRV could achieve a better performance of VNS outcome prediction and, therefore, help patients who are not suitable for VNS to avoid the high cost of surgery and possible risks of long-term stimulation.

## Introduction

More than 65 million people affected by epilepsy worldwide and 10–50% of patients with drug-resistant epilepsy (DRE) can potentially take advantage of curative epileptic surgery through craniotomy after complete preoperative evaluations (1–3). Vagus nerve stimulation (VNS) therapy is a beneficial option for patients who are not suitable for craniotomy or cannot benefit from craniotomy. As an adjuvant therapy for patients with DRE, VNS has been widely used by more than 100,000 patients worldwide (4). Despite the increasing application, studies have shown that VNS can rarely help patients achieve complete seizure freedom. Specifically, seizures were reduced by 50% or more in ~50% of the patients, while about a quarter of patients with DRE do not get any benefit from the VNS therapy (5). The outcomes of VNS in patients vary greatly. This may be due to the complexity of clinical factors, including etiology syndromes, etiology, and usage of antiepileptic drugs (AEDs) (6). Therefore, presurgical identification of patients who are not suitable for VNS is valuable.

Previous studies have explored the relationship between preoperative heart rate variability (HRV) indices and VNS outcome and evaluated the feasibility of HRV indices to be used as predictors for the response to VNS (7–9). Liu et al. suggested that presurgical HRV measurements representing parasympathetic cardiac control were significantly associated with the responsiveness to VNS, and found that non-responders (<50% reduction in seizure frequency at 1-year follow-up) had significantly lower root mean square of the differences between adjacent RR intervals (RMSSD), percentage of differences between successive NN intervals above 50 ms (pNN50), high frequency (HF), and SD1 than responders. Among them, RMSSD had the greatest discriminatory power (AUC: 0.774 ± 0.063) in the result of receiver operating characteristic curve analysis (7, 8). However, Hödl et al. came to the contradictory conclusion that non-responders had significantly higher HF than responders before surgery and at 1-year follow-up (9). In the above studies, significance test methods were performed to analyze the importance of each HRV variable to efficacy. Significant test depends on sample size and does not give any indication of the relevance between variables (10, 11). Due to these limitations, based on single HRV indices, the generalization ability of VNS outcome prediction models might be poor. There were increasing uses of multivariate indicators that could better quantify the magnitude of difference (12). Compared with traditional statistical methods, machine learning could review large volumes of data and discover specific trends and patterns that would not be apparent to humans. Through random forest (RF) classifier, the normalization of data is not required and the risk of overfitting can be reduced (13, 14). Machine learning has been widely used in the medical field in recent years. In addition, researchers had applied this method to classify response to chronic VNS on the basis of intrinsic connectivity within thalamocortical circuitry (15–17). Combining machine learning and HRV indices might provide a simpler and more conducive way to the prediction of VNS outcome.

Using traditional HRV analysis, previous studies found that autonomic activity shows a circadian rhythm with a prevalence of sympathetic tone during the day and a considerable relative increase in parasympathetic tone during the night (18–20). Ronkainen et al. measured the interictal circadian rhythm of HRV in 17 patients with DRE and found that they had decreased 24-h diurnal fluctuations of low frequency (LF) and HF compared with healthy control (21). Furthermore, studies have shown that VNS increased the complexity of HRV with DRE patients during sleep state and decreased it during awake state (22, 23). Since VNS has potential effects on cardiac autonomic nerve function, the circadian variation of preoperative HRV might also be related to the performance of efficacy prediction. Therefore, it is worthwhile to investigate the influence of HRV differences in the sleep and awake states on the prediction of VNS outcome. In this paper, we propose a VNS outcome prediction method with RF classifier for patients with DRE based on preoperative HRV indices. HRV analysis was performed on both patients and healthy controls. Results obtained in sleep and awake states were further compared and feature selection was performed to improve the statistical model accuracy.

## Materials and Methods

### Participants and Study Design

Fifty-nine DRE patients were implanted with VNS equipment (G111, Pins Medical Ltd., Beijing, China) between 2014 and 2015 and underwent 1-year follow-up evaluation. Seven centers took part in this study, namely Beijing Tiantan Hospital Capital Medical University, Sanbo Brain Hospital Capital Medical University, TsingHua University YuQuan Hospital, Peking University First Hospital FengTai Hospital, Chinese PLA General Hospital, First Affiliated Hospital of PLA General Hospital, and Navy General Hospital. All patients underwent a complete preoperative evaluation, including 24-h ECG recordings; long-term video-EEG, MRI, or PET; and comprehensive clinical assessments. The inclusion criteria were as follows: (1) 7–60 years old; (2) in good health except for the epilepsy; (3) at least one seizure per month; (4) have been tested at least two suitable AEDs for tolerance or blood level at the upper limit of the target range, and at least two of them can be tolerated at normal dose; and (5) minimum mental state examination (MMSE) score ≥ 18 (no severe cognitive impairment). Exclusion criteria were as follows: (1) results of MRI or PET show the epilepsy was caused by intracranial space-occupying lesions; (2) tumor, cardiopulmonary anomaly, diabetes, progressive neurological disease, asthma, mental disease, and other surgical contraindications; (3) smoking, alcohol addiction, and breathing disorders related to sleep; and (4) a history of medication which may affect the autonomic function. Healthy controls were selected in accordance with the gender ratio and age range of the DRE patients. All healthy controls had no medication or other disease affecting the function of the autonomic nerve system based on their medical history and physical examination results. During the 3 months before VNS surgery and the 1-year follow-up period after VNS surgery, the number and dose of AED regimens remained unchanged. Our study was approved by the Institutional Review Committee of Beijing Tiantan Hospital Capital Medical University. All subjects or their parents gave written and informed consent including the collection of their information and usage in our research.

### ECG Recording and Preprocessing

A 12-lead, consecutive 24-h ECG recording was performed under free-moving condition by Holter monitoring system (MIC12H-3S, JincoMed, Beijing, China) at a digital sampling rate of 500 Hz for all participants. During the recording, patients were asked to document the type, time, and duration of their daily activities and possible seizures. After ECG records were visually checked for potential non-sinus or ectopic beats by a PC-based acquisition system (SkyHolter, JincoMed), we extracted RR intervals from the stable signal provided by lead V5 and eliminated ectopic beats by interpolation based on surrounding normal beats for adjustment (8). Based on the HR characteristics, we removed the effects of seizures periods and reduced the variability of physical activities on the experimental results, by selecting 4-h periods of reliable RR intervals in quiet awake and sleep states of each patient for further analysis (24, 25).

### HRV Analysis

HRV analysis was performed on 4-h RR intervals by NeuroKit2 (26), which is a toolbox based on Python 3.6.3 for comprehensive HRV analysis with a total of 52 indices. Time domain, frequency domain, and non-linear HRV indices were measured according to the guidelines and studies on the HRV assessment and spectral analysis techniques (27–33). Time domain indices included the RMSSD, mean RR intervals (MeanNN), standard deviation of the RR intervals (SDNN), standard deviation of differences between adjacent RR intervals (SDSD), percentage of differences between successive NN intervals above 50 /20 ms (pNN50/pNN20), SDNN divided by MeanNN (CVNN), median absolute deviation of the RR intervals (MadNN), MadNN divided by the median of the absolute differences of their successive differences (MCVNN), interquartile range of the RR intervals (IQRNN), and baseline width of the RR intervals distribution obtained by triangular interpolation (TINN). Spectral analysis by using fast Fourier transform (FFT) included frequency components as follows: very low frequency (VLF; 0.003–0.04), LF (0.04–0.15 Hz), HF (0.15–0.4 Hz), and normalized LF (LFn) and HF (HFn), obtained by dividing the low frequency power by the total power. Non-linear HRV metrics contain the characteristics of the Poincaré plot geometry, indices of heart rate asymmetry, indices of heart rate fragmentation, and indices of complexity (31, 32). Among them, SD1 is an index of short-term RR interval fluctuations, while SD1d and SD1a are short-term variance of contributions of decelerations (prolongations of RR intervals) and accelerations (shortenings of RR intervals), respectively (33). Contrary to SD1, SD2 represents long-term RR interval fluctuations, so as SD2d and SD2a. S is the area of ellipse described by SD1 and SD2. The cardiac sympathetic index (CSI) is calculated by dividing the longitudinal variability of the Poincaré plot by its transverse variability, and slope index (SI) described the asymmetry of the Poincaré plot. PSS is the percentage of NN intervals in short segments. Among all the HRV metrics, LF component reflects the dual regulation of sympathetic and vagus nervous system, and VLF is possibly related to sympathetic activity (27, 28). In addition, studies have shown that in the case of low-frequency breathing, the regulation of vagal tone to heart rate will significantly affect the LF power (27, 34, 35). At present, it is agreed that VLF is an intrinsic rhythm of the heart and the basis for the human body to maintain a healthy state. Under normal circumstances, VLF in the resting state can reflect the regulation of sympathetic nervous system on heart rate (35, 36).

### Statistical Analysis

The Mann–Whitney *U*-test was performed to compare the demographic data and HRV indices of responders and non-responders, and data were presented as mean ± standard deviation or number (percentage). Fisher's exact tests or chi-square tests were applied for qualitative or categorical variables between different groups. Features with a threshold of *p* < 0.05 were considered statistically significant and reserved for further selection.

### VNS Outcome Prediction With Feature Selection

After HRV indices of all patients were analyzed by NeuroKit2, we applied RF algorithm with 20 trees to train the prediction model. Feature selection was applied before training to improve the prediction performance. In contrast to other dimensionality reduction techniques, feature selection does not alter the original form of the variables but select a subset of them (37). Specifically, in our study, feature selection removed HRV indices irrelevant with VNS outcome, thus reducing the difficulty of classification tasks and decreased the risk of overfitting.

We tried both univariate feature filter and recursive feature elimination (RFE) algorithm for feature selection. Univariate filter technique assesses the relevance of each feature by analyzing the intrinsic properties of the data and needs to be performed only once for classifier evaluation (37). Specifically, we chose chi-squared statistics as measurement for the relevance between variables and targets, for it was a relevance filtering method specifically for discrete targets (i.e., classification problems). The importance of the whole feature subsets was determined by the sum of relevance score of each feature and low-scoring features were removed. In order to evaluate the prediction performance of univariate filter, we performed the fast correlation-based feature filter (FCBF), a kind of multivariate filter approach as a comparison. FCBF selects features with high correlation with the target and little correlation with other variables through a metric called symmetrical uncertainty (38). In addition to filter algorithm, we tried RFE, a kind of wrapper algorithm that embeds model hypothesis search in feature subsets search. Unlike the filter method, the RFE algorithm directly uses the performance of the final model as the evaluation criterion for feature selection, in other words, to randomly search the feature subset with the best prediction results for the given model. Specifically, for a feature set with a number of *d*, we calculate the error of all feature subsets (2^*d*^−1) from their cross-validation scores and select the subset with the smallest error as the final result. To avoid choosing dominant features among few subjects, we applied nested cross-validation (CV), where an inner CV loop is used to perform the tuning of the parameters, while an outer CV is used to compute an estimate of the error (39). Nested cross-validation can tune decoders' parameters while avoiding circularity bias. We performed nested CV with *k*-fold set to 5 both in the inner and outer loops (40). In order to obtain a stable sorted result of all HRV indices according to their relevance score, we repeated the RFE process 50 times and accumulated the ranking of each index during each experiment. While the computation through the univariate filter method was simpler, it ignored the interaction with the classifier and the dependences between features, for the search space of the optimal solution was only performed in the feature subset. On the other hand, the RFE method costed a relatively large amount of computation but often brought good performance (37, 38).

After the sorted HRV indices were obtained through the above methods, respectively, we chose different numbers of top-ranked indices as feature subsets for VNS outcome prediction. A leave-one-out (LOO) cross-validation was carried out, which was a *K*-fold cross-validation with *K* equal to the number of subjects in the dataset. For each time of validation, one subject was taken as the test set for prediction and the function approximator is trained based on the rest of the subjects. Same as *K*-fold cross-validation, the average error is computed to evaluate the model. Although it was a computationally expensive procedure to perform, the LOO technique was suitable for small datasets and contributed to a relatively reliable and unbiased evaluation of the classification model (41). At last, we could obtain a LOO cross-validated selection of the best feature subset corresponding to the classification scores.

## Results

### Participants

A total of 59 patients with DRE and 50 healthy controls participated in our study. Patients included 40 males and 19 females and healthy controls included 34 males and 16 females. Both groups range in age from 7 to 38 years old. Demographic data of all the participants are presented in Supplementary Table 1. No significant differences were presented between DRE patients and healthy controls. At 1-year follow-up evaluation, 30 patients responded to VNS therapy and had monthly seizure frequency decreased by more than 50% (responders). Eight patients were seizure-free at the end of 1-year VNS treatment. Demographics and clinical factors of 59 DRE patients are listed in Table 1. We observed no significant differences between responders and non-responders in demographic data, seizure characteristics, ictal scalp EEG characteristics, AED information, etiology, and VNS settings (all *p* > 0.05). No serious adverse events were found in the participants.

**Table 1:** Preoperative clinical data and VNS settings at 1-year follow-up of responders and non-responders.

**Variables**	**Responders (*n* = 30)**	**Nonresponders (*n* = 29)**	***p*-value**
Demographic data			
Age (years)	19.6 ± 7.9	18.8 ± 8.3	0.824
Male/female	23/7	17/12	0.170
BMI (kg/m^2^)	22.5 ± 4.3	22.2 ± 4.3	0.544
Seizure characteristics, no. (%)			
Epilepsy duration (years)	12.4 ± 7.4	10.6 ± 7.1	0.475
Seizure per month	90.3 ± 176.4	59.4 ± 89.1	0.164
FS	7 (23.3%)	3 (10.3%)	0.299
GS	11 (36.7%)	8 (27.6%)	0.580
FS + GS	12 (40.0%)	18 (62.1%)	0.120
Cerebral lesions (ictal scalp EEG), no. (%)			
Temporal	17 (56.7%)	19 (65.5%)	0.596
Frontal	11 (36.7)	7 (24.1%)	0.399
Parietal	7 (23.3%)	10 (34.5%)	0.399
Occipital	3 (10.0%)	8 (27.6%)	0.104
Non-specific EEG abnormalities	9 (30.0%)	7 (24.1%)	0.771
Number of AEDs	3.0 ± 1.2	3.0 ± 1.0	0.848
Etiology (MRI), no. (%)			
Symptomatic	13 (43.3%)	12 (41.4%)	1.000
Cryptogenic	17 (56.7%)	17 (58.6)	1.000
VNS settings			
Current amplitude (mA)	1.4 ± 0.6	1.5 ± 0.4	0.619
Pulse width (μs)	441.7 ± 105.7	431.0 ± 111.7	0.525
Frequency (Hz)	29.5 ± 1.5	28.8 ± 3.9	0.415
VNS on time (s)	30.0 ± 0.0	29.7 ± 1.6	0.161
VNS off time (min)	5.0 ± 0.0	5.7 ± 3.6	0.161

*BMI, body mass index; FS, focal seizure; GS, generalized seizure; EEG, electroencephalography; AEDs, antiepileptic drugs; MRI, magnetic resonance imaging; VNS, vagus nerve stimulation*.

### HRV Differences Between Sleep and Awake States

Fifty-two presurgical HRV indices including time domain, frequency domain, and non-linear measurements were performed on participants in sleep and awake states, respectively. DRE patients had significantly lower RMSSD, MeanNN, SDNN, SDSD, MedianNN, MadNN, IQRNN, pNN50, pNN20, TINN, VLF, LF, HF, VHF, SD1, SD2, S, CSI Modified, SD1d, SD1a, SD2d, SD2a, SDNNd, and SDNNa in comparison with the healthy controls (Supplementary Table 2). For the differences of HRV indices between sleep and awake states, the results of significance tests showed that in sleep state 30 indices with responders and 21 indices with non-responders were significantly higher than those in awake state (Supplementary Table 3). For the differences between responders and non-responders, in sleep state, 35 indices of responders were significantly higher than those of non-responders except for CSI (3.59 ± 1.7 vs. 4.29 ± 1.35, *p* = 0.005) and SI (49.99 ± 0.02 vs. 50.0 ± 0.01, *p* = 0.003) (Table 2), while in awake state, 16 indices of responders showed the same significance (RMSSD, MeanNN, SDSD, CVSD, MedianNN, pNN50, pNN20, VLF, LF, HF, LnHF, SD1, SD1d, SD1a, ApEn, SampEn), which were included in the 35 indices. Comparison with *p* < 0.05 was considered statistically significant, and the following HRV indices showed significant differences between responders and non-responders in sleep state but not in awake state including SDNN (*p* = 0.005 vs. *p* = 0.306), CVNN (*p* = 0.008 vs. *p* = 0.395), MadNN (*p* = 0.001 vs. *p* < 0.497), MCVNN (*p* = 0.002 vs. *p* = 0.204), IQRNN (*p* = 0.001 vs. *p* < 0.497), TINN (*p* = 0.039 vs. *p* = 0.431), VHF (*p* = 0.001 vs. *p* = 0.083), SD2 (*p* = 0.011 vs. *p* = 0.285), SI (*p* = 0.003 vs. *p* = 0.290), SD2d (*p* = 0.009 vs. *p* = 0.344), SD2a (*p* = 0.010 vs. *p* = 0.306), SDNNd (*p* = 0.004 vs. *p* = 0.311), and SDNNa (*p* = 0.005 vs. *p* = 0.295).

**Table 2:** Preoperative time domain, frequency domain, and non-linear HRV indices of responders and non-responders.

**HRV indices**	**Sleep**			**Awake**		
	**Responders**	**Non-responders**	***P*-value**	**Responders**	**Non-responders**	***P*-value**
RMSSD	54.5 ± 26.2	34.9 ± 15.0	<0.001	27.0 ± 13.1	21.6 ± 9.8	0.025
MeanNN	896.4 ± 124.6	835.5 ± 138.3	0.031	673.3 ± 85.6	630.5 ± 87.4	0.041
SDNN	87.0 ± 26.3	70.7 ± 21.6	0.005	70.6 ± 21.6	68.4 ± 24.0	0.306
SDSD	54.5 ± 26.2	34.9 ± 15.0	<0.001	27.0 ± 13.1	21.6 ± 9.8	0.025
CVNN	0.10 ± 0.02	0.08 ± 0.02	0.008	0.10 ± 0.02	0.11 ± 0.03	0.395
CVSD	0.06 ± 0.03	0.04 ± 0.02	0.001	0.04 ± 0.02	0.03 ± 0.01	0.052
MedianNN	902.2 ± 130.8	842.0 ± 143.5	0.034	672.9 ± 86.4	629.1 ± 88.6	0.036
MadNN	76.7 ± 29.9	56.5 ± 14.7	<0.001	69.9 ± 25.9	71.7 ± 30.7	0.497
MCVNN	0.08 ± 0.03	0.07 ± 0.01	0.002	0.10 ± 0.03	0.11 ± 0.04	0.204
IQRNN	105.4 ± 43.4	77.0 ± 20.4	<0.001	96.2 ± 35.2	98.2 ± 43.0	0.497
pNN50	30.2 ± 17.3	14.9 ± 13.4	0.001	7.94 ± 8.79	3.75 ± 5.48	0.014
pNN20	60.1 ± 19.1	46.8 ± 18.2	0.002	31.3 ± 17.3	19.8 ± 13.7	0.006
TINN	690.9 ± 160.7	631.6 ± 185.9	0.039	577.8 ± 115.5	587.0 ± 169.3	0.431
VLF	2079.9 ± 1566.0	1423.0 ± 958.1	0.019	1338.4 ± 848.5	1020.0 ± 866.0	0.026
LF	1569.3 ± 1, 214.9	1097.3 ± 759.9	0.037	715.6 ± 496.4	593.5 ± 653.6	0.047
HF	832.4 ± 717.4	525.4 ± 360.3	0.010	490.8 ± 369.5	357.5 ± 361.8	0.041
VHF	119.6 ± 83.8	62.1 ± 53.7	0.001	76.2 ± 63.5	66.6 ± 108.6	0.083
LnHF	6.42 ± 0.82	5.97 ± 0.86	0.010	5.88 ± 0.85	5.51 ± 0.83	0.041
SD1	38.5 ± 18.5	24.7 ± 10.6	<0.001	19.1 ± 9.2	15.3 ± 7.0	0.025
SD2	116.1 ± 34.7	96.5 ± 29.7	0.011	97.7 ± 29.9	95.4 ± 33.6	0.285
SD1SD2	0.33 ± 0.12	0.26 ± 0.09	0.005	0.20 ± 0.07	0.16 ± 0.04	0.052
S	15382.1 ± 12014.7	8133.5 ± 5, 719.7	<0.001	6383.8 ± 4516.9	5080.9 ± 4029.3	0.105
CSI	3.59 ± 1.70	4.29 ± 1.35	0.005	5.86 ± 2.17	6.67 ± 2.02	0.052
CVI	4.78 ± 0.34	4.52 ± 0.30	<0.001	4.41 ± 0.31	4.32 ± 0.30	0.105
IALS	0.51 ± 0.05	0.49 ± 0.05	0.041	0.49 ± 0.06	0.47 ± 0.05	0.076
PSS	0.78 ± 0.11	0.73 ± 0.1	0.028	0.74 ± 0.08	0.71 ± 0.08	0.087
SI	49.99 ± 0.02	50.0 ± 0.01	0.003	50.0 ± 0.02	50.0 ± 0.03	0.290
SD1d	27.9 ± 13.7	17.7 ± 7.6	<0.001	13.6 ± 6.6	11.0 ± 5.2	0.030
SD1a	26.5 ± 12.5	17.2 ± 7.4	0.001	13.4 ± 6.5	10.6 ± 4.6	0.026
SD2d	77.9 ± 21.7	65.2 ± 19.9	0.009	68.6 ± 20.6	67.0 ± 23.4	0.344
SD2a	86.0 ± 27.2	71.1 ± 22.3	0.010	69.6 ± 21.9	67.9 ± 24.2	0.306
SDNNd	58.9 ± 16.9	47.9 ± 14.5	0.004	49.6 ± 14.9	48.1 ± 16.7	0.311
SDNNa	64.0 ± 20.2	51.87 ± 16.1	0.005	50.2 ± 15.8	48.6 ± 17.3	0.295
ApEn	1.44 ± 0.27	1.32 ± 0.20	0.007	1.07 ± 0.3	0.89 ± 0.22	0.010
SampEn	1.31 ± 0.27	1.20 ± 0.20	0.008	0.89 ± 0.28	0.71 ± 0.21	0.009

*Data were presented as mean value ± standard deviation*.

### VNS Outcome Prediction With Feature Selection

**Figure 1:**
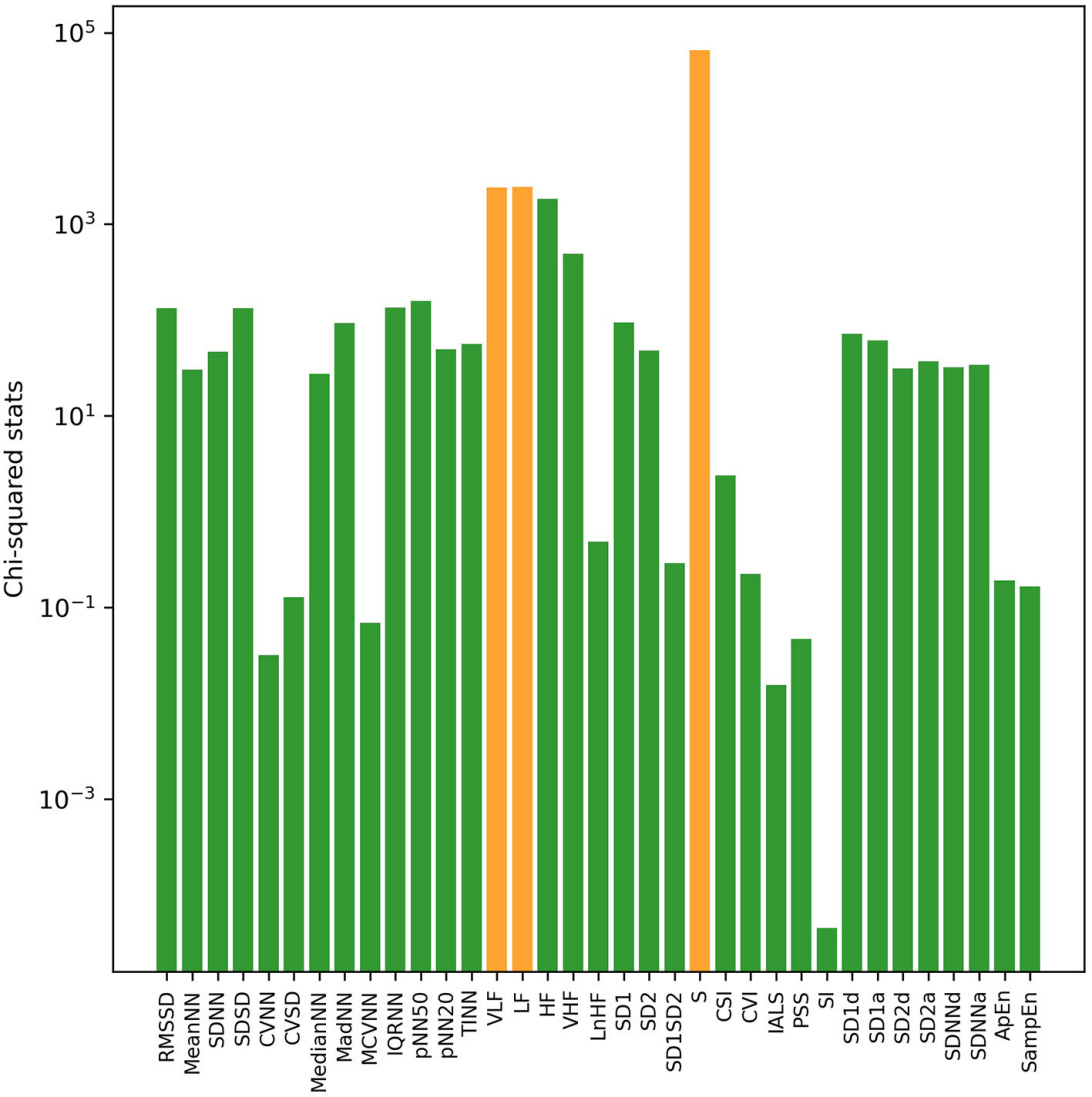
The log transformation of chi2-squared statistics of each heart rate variability (HRV) index in sleep state computed by the univariate filter method. The top three HRV indices are shown in orange bars.

**Figure 2:**
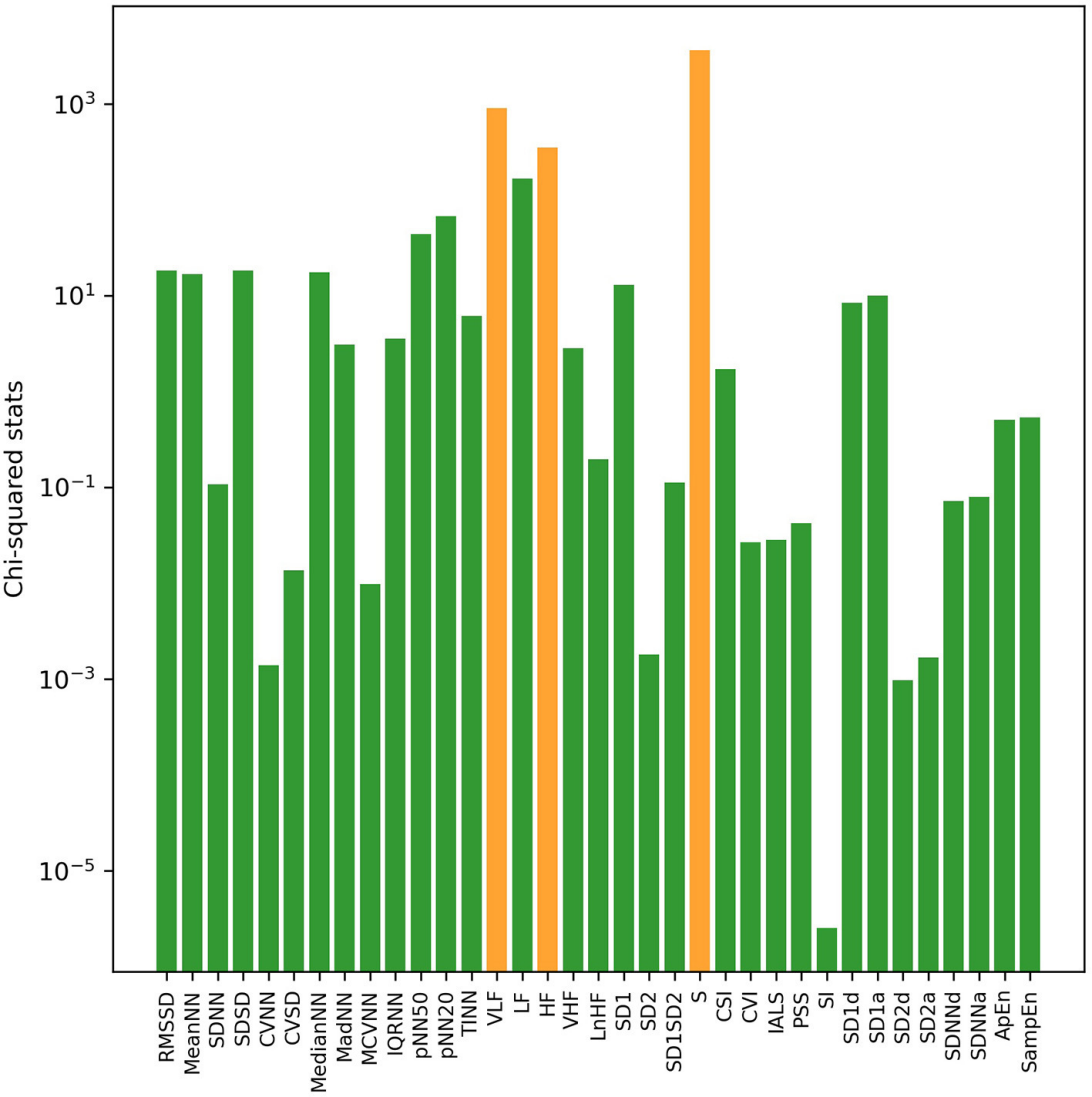
The log transformation of chi2-squared statistics of each HRV index in awake state computed by the univariate filter method. The top three indices are shown in orange bars.

With univariate filter and RFE methods, sorted results of HRV indices based on their relevance with VNS efficacy in both sleep and awake states were obtained, respectively. Through calculation of chi-squared statistics by the univariate filter method, the top three indices were S, LF, and VLF in sleep state (Figure 1) and S, VLF, and HF in awake state (Figure 2). In both states, SI ranked last with the smallest chi-squared score, which presented the weakest relevance with the VNS outcome. With the RFE method and nested cross-validation, accumulated ranking results of importance score of each HRV indices are shown in Supplementary Figures 1, 2. After 50 iterations of algorithms, LF ranked first steadily in both states (got the smallest cumulative results), indicating that through nested cross-validation, it did the greatest contribution to the classification of responders and non-responders, and it was followed by PSS and S in sleep state and pNN20 and VLF in awake state, respectively.

**Figure 3:**
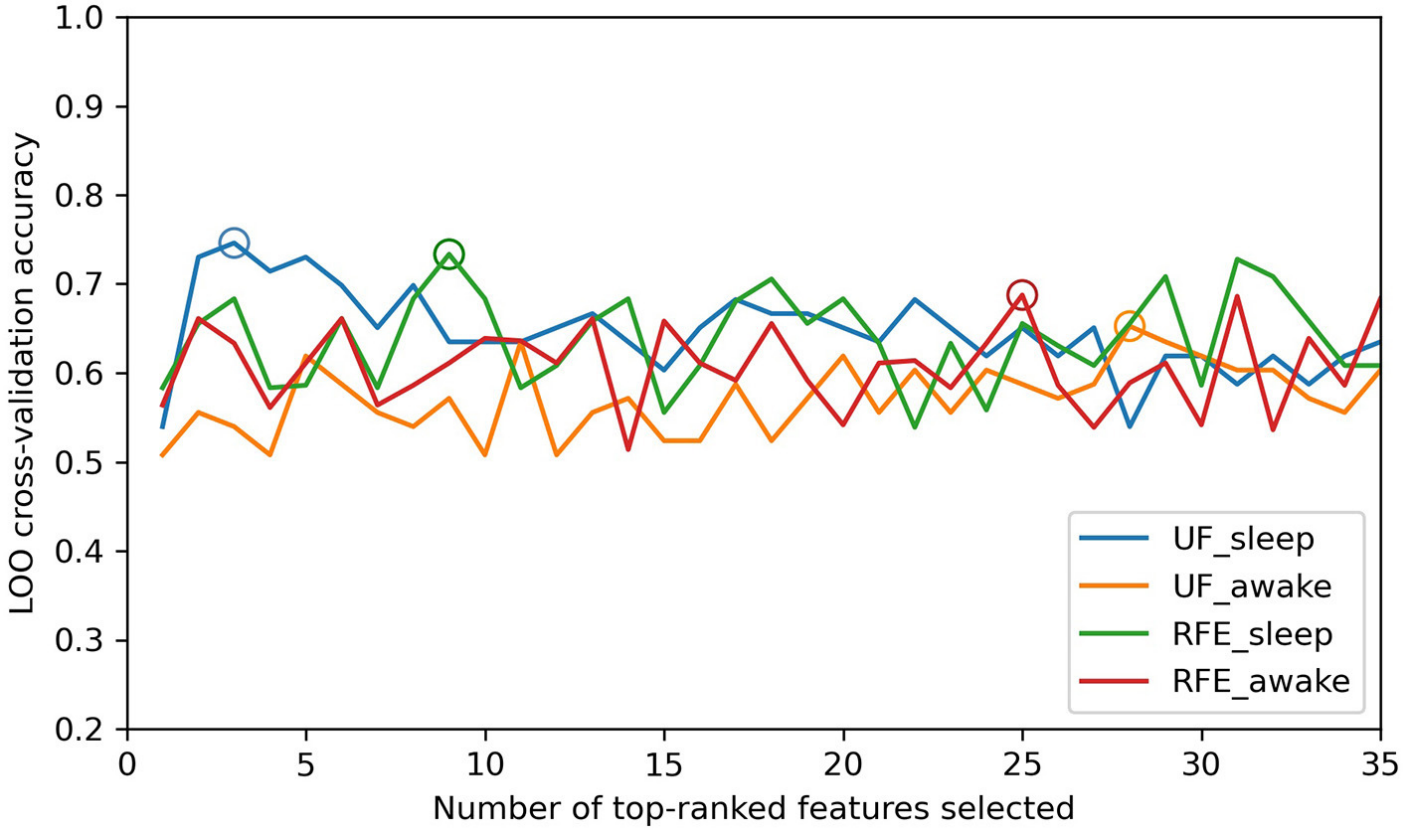
The distribution of prediction accuracy with different numbers of top-ranked HRV indices as features. UF-sleep, univariate filter with data in sleep state; UF-awake, univariate filter with data in awake state; RFE-sleep, RFE with data in sleep state; RFE-awake, RFE with data in awake state. The best classification results with optimal size of features are depicted by circles (UF_sleep: 3, 74.6%; RFE_sleep: 9, 73.4%; UF_awake: 28, 65.3%; RFE_awake: 25, 68.8%).

The distribution of prediction accuracy with a number of top-ranked HRV indices as features in both sleep and awake states is shown in Figure 3, and performances with optimal feature combination are described in Table 3. The best prediction performance was obtained by the univariate filter method with data in sleep state when selecting the top three HRV indices (Figure 3), achieving 74.6% accuracy, 80.0% precision, 70.6% recall, and 75.0% F1 score. Compared with the awake state, the best prediction performance in sleep state was better and required fewer top-ranked HRV indices for both feature selection methods. The prediction performances of univariate filter with other relevance measurements (ANOVA *F*-value, mutual information) and multivariate filter method (FCBF) are presented in Supplementary Table 4. The best performance of univariate filter among all the metrics was 74.6% with chi-squared statistics, while mutual information presented the worst results (69.8% accuracy in sleep state and 65.1% accuracy in awake state). Based on chi-squared statistics, univariate filter performed better than the multivariate filter approach. Other classifiers were also compared with RF for the outcome predictions (Supplementary Table 5). The RF classifier with univariate filter in sleep state presented the best prediction results (five-fold cross-validation: 78.3% accuracy, LOO: 74.6% accuracy). In addition, with the RF classifier, the model achieved best performances in the combinations of different states, feature selection methods, and cross-validation methods, except for the result based on RFE and LOO methods in awake state which was slightly lower than SVM (RF: 64.9% accuracy, SVM: 66.7% accuracy). The lowest result was obtained in the classification using KNN with the univariate filter method in awake state (five-fold cross-validation: 58.5% accuracy, LOO: 58.7% accuracy).

**Table 3:** VNS outcome classification performances of univariate filter and RFE feature selection methods in sleep and awake states.

**States**	**Univariate filter**				**RFE**			
	**Accuracy (%)**	**Precision (%)**	**Recall (%)**	**F1 (%)**	**Accuracy (%)**	**Precision (%)**	**Recall (%)**	**F1 (%)**
Sleep	74.6	80.0	70.6	75.0	73.4	80.3	86.4	77.9
Awake	65.3	66.4	70.5	68.4	68.8	73.7	76.4	69.5

## Discussion

Our study retrospectively investigated the presurgical HRV indices of 59 DRE patients and 50 healthy controls. To study if HRV would be a true biomarker for VNS outcome related to abnormal autonomic nerve function of DRE patients, we provided a comparison to a matched normal population and found that 49 indices showed significant differences between DRE patients and healthy controls. Among them, 25 indices of healthy controls were significantly higher than those of DRE patients, indicating that DRE patients showed a cardiac autonomic function response as a significant decrease of HRV indices (7). HRV indices in sleep state were more conducive to distinguish between responders and non-responders and significantly higher than indices obtained in awake state. Univariate filter and RFE algorithms were performed to select the optimal feature set and sort HRV indices based on their relevance to VNS outcome for 1-year follow-up. Combining HRV indices in sleep state as feature vector based on data attributing importance ranking, the statistical model of the RF classifier achieved better prediction performance, and the optimal size of the feature set obtained by the univariate filter method achieved the best prediction performance. To the best of our knowledge, this is the first study that combines a machine learning method with preoperative HRV indices for VNS outcome prediction, and investigates the effect of sleep and awake states on prediction performance.

Our findings showed higher presurgical HRV of patients with DRE in sleep state than in awake state, which was consistent with previous studies (42–44). The overall trends of the circadian rhythm of heart rate and HRV are opposite. Specifically, heart rate in sleep state is lower than in awake state. About an hour before awakening, heart rate gradually rises and maintains a relatively stable level in awake state. Meanwhile, the time and frequency domain indices of HRV in sleep state are higher and gradually decline with the end of sleep, dropping to the lowest value in a fully awake state. The diurnal change of HRV indicates the characteristic of stronger vagal tone in sleep state. The circadian rhythm of HRV was closely related to the cardiac autonomic nervous system, reflecting the dynamic balance of the mutual influence of the sympathetic and parasympathetic nervous system.

Importantly, our results suggested better performance of presurgical data in sleep state on differentiating responders and non-responders based on significance test. Moreover, the prediction accuracy with the optimal feature set of data in sleep state was better than that in awake state for both feature selection methods. Compared with awake state, more HRV indices showed significant differences between responders and non-responders in sleep state, which might be related to the stronger external interference in awake state on ECG recordings from the environment, emotions, daily activities, and other factors. The specific reasons are still unclear, since the circadian rhythm of HRV is also affected by age, gender, drugs, and various diseases (45). Besides that, studies have shown that VNS has the potential to restore the natural chaotic behavior of the heart by activating the parasympathetic division of the autonomic nervous system and to increase the complexity of HRV during sleep and decrease it during wakefulness (22, 23). Patients who experienced more than 50% reduction in seizures after 1 year of VNS treatment had a significantly higher amount of deep sleep (non-rapid eye movement 3, NREM3) (9, 46). The hypnotic agent adenosine has the potential of driving deep sleep and is anti-epilepsy. Therefore, adenosine receptor expression and signaling might also participate in the mechanism of VNS (46). In addition, in our research, responders showed significantly higher presurgical HRV indices including RMSSD, pNN50, SD1, and HF, which agreed with previous findings (7). Since the HF component mainly reflects the vagal activity and the RMSSD, pNN50, and SD1 are high correlates with HF (8), it was indicated that patients with preoperative cardiac autonomic nerve function damage, especially for severe reduction of vagal tone, have a poor postoperative prognosis of VNS. Possible factors contributed to the higher vagal activity of responders including the effects of the long-term intake of AEDs, psychological comorbidity, and recurrent seizures (34, 47).

Importance ranking results of HRV indices were obtained by univariate filter and RFE feature selection methods. In the results of univariate filter, S ranked first in both states, followed by LF and VLF, while the RFE method ranked LF in first place. Among the top-ranked non-linear indices, S describes the area of ellipse in Poincaré plot and correlates with RMSSD and baroreflex sensitivity, which is the change in interbeat interval duration per unit change in blood pressure (29). Apart from frequency domain HRV indices, heart rate complexity (ApEn, SampEn) and fragmentation (PSS) indices also got high importance scores in the RFE ranking results. Both feature selection methods emphasized the importance of LF and non-linear HRV indices to VNS outcome prediction. ApEn measures the regularity and complexity of time series. While large ApEn values indicate low predictability of fluctuations in successive RR intervals, small ApEn values mean that the signal is regular and predictable (35). SampEn was designed to provide a less biased and more reliable measure of signal regularity and complexity (36). As for frequency domain indices, LF mainly reflects the activity of the baroreceptors in the resting state, and it would be significantly affected by the regulation of the vagal tone under low respiratory rate (34–36). Because vagal tone is affected by respiratory cycle, stress, anxiety, worry, and other emotions, LF could also be indirectly influenced by these external factors. Previous studies have pointed out that VLF was the inherent rhythm of the heart to maintain the health of the body, correlated with the change in body temperature and vasomotor tone, and affected the regulation of the renal angiotensin system (27, 34). Similar to LF, in the resting state, VLF could reflect the regulating effect of sympathetic nerves on heart rate. Since data in sleep state were more beneficial to distinguish VNS outcome, in sleep state, the optimal feature combinations for the best prediction performance are three and eight top-ranked HRV indices by the univariate filter and RFE method, respectively, while in awake state, the optimal feature combination required more HRV indices (univariate filter: 28 indices, RFE: 26 indices).

The present studies on VNS outcome prediction also investigated the seizure characteristics and demographic information of patients with DRE and conducted discussions on the analysis of physical signs such as MRI, EEG, and ECG (7, 8, 48–53). Whether seizure characteristics had potential relationship with the response of VNS is still uncertain. Although epileptic seizures were rarely completely controlled, generalized epilepsy benefited more than partial seizures from VNS therapy (54). Another study demonstrated that patients with predominantly partial seizures responded most favorably to VNS, whereas those with generalized tonic–clonic seizures responded least favorably (5). The study of predicting the VNS efficacy based on presurgical EEG computed the power spectral analyses retrospectively on preoperative EEG recordings from 60 epileptic patients with VNS, indicating that there were significant differences in EEG reactivity between responders and non-responders (55). Specifically, the dynamics of alpha and gamma activity were strongly related to the VNS outcome. Although most ECG studies on cardiac function of patients with DRE have focused on heart rate-related changes, there are still a range of other variables of ECG that have potential value for epilepsy detection and seizure prediction. Studies have shown that the information of seizure contained in the single-channel ECG is comparable with that of scalp EEG, and the entire spectrum of ECG is beneficial for the assessment of patient's preseizure state (56). Another work reported on the ECG changes of epilepsy patients during preictal, ictal, interictal, and postictal states (57). It suggested that patients were more likely to have abnormal QTc intervals and ST segments, elevated T waves, increased P wave dispersion, and prolonged PR intervals during the interictal period. Changes during preictal and ictal states including arrhythmia, prolonged QTc intervals, and ST segment abnormalities are also reported. Those wave characteristics of ECG might have potential relevance to cardiac function and be useful to the VNS outcome prediction in the future.

Compared with a previous work, some novelties are shown in this paper. Through machine learning and HRV indices, a multivariate statistical model for predicting the VNS outcome was obtained and the importance of each HRV index in outcome prediction was measured by feature selection. LOO validation, which was suitable for a small dataset, was performed to contribute to a reliable and unbiased evaluation of the classification model. In order to study the influence of circadian rhythm on the prediction of VNS outcome, we compared the HRV indices and prediction performances in different states and found the data in sleep state showing better outcome predictive power. In addition, more time domain, frequency domain, and non-linear HRV indices were included in this paper compared with previous work (7, 8), so as to provide a more comprehensive basis for the VNS outcome prediction model.

Several limitations are present in our study. Although we excluded the possible influence of demographic data and other clinical examination results on VNS outcome prediction, potential effects including respiratory rate, type of seizure, and different types and dosages of AEDs on heart rate and HRV were not completely elucidated. We obtained 4-h RR intervals in awake state by selecting the periods when the heart rate is relatively stable, which might not exclude the interference from mild exercises such as walking to our experimental results. Besides, we recruited DRE patients with a wide age range, and the potential influence of age on VNS outcome prediction should be investigated in the future. To further establish the wide application of ECG-based VNS outcome prediction in clinical use, a sizeable, multicenter, and prospective study is required to evaluate the feasibility and reliability of the VNS outcome prediction model.

## Conclusion

In conclusion, based on the preoperative HRV and machine learning method, our statistical model suggested that ECG recorded in sleep state could achieve better prediction performance of VNS outcome. Both univariate filter and RFE feature selection methods emphasized the importance of LF component and non-linear indices to efficacy prediction. These findings are beneficial for patients to evaluate whether VNS surgery is suitable for them and for researchers to further investigate the influence and effect of circadian rhythm of HRV on efficacy prediction.

## Data Availability Statement

The original contributions presented in the study are included in the article/Supplementary Material, further inquiries can be directed to the corresponding author/s.

## Ethics Statement

The studies involving human participants were reviewed and approved by the Institutional Review Committee of Beijing Tiantan Hospital Capital Medical University. Written informed consent to participate in this study was provided by the participants' legal guardian/next of kin. Written informed consent was obtained from the individual(s), and minor(s)' legal guardian/next of kin, for the publication of any potentially identifiable images or data included in this article.

## Author Contributions

XF, Z-YW, C-HH, and H-WH conceived and designed the experiments. F-GM, Y-GG, Y-SM, S-LL, J-LL, and M-MZ diagnosed and carried out the VNS surgery for the patients with DRE. H-YL, ZY, and T-YC collected and analyzed the ECG data. L-ML made scientific comment on the manuscript. All authors contributed to the article and approved the submitted version.

## Conflict of Interest

H-WH, C-HH, and L-ML report personal fees from Beijing Pins Medical Co., outside the submitted work. The remaining authors declare that the research was conducted in the absence of any commercial or financial relationships that could be construed as a potential conflict of interest.

## References

[1] Moshé SL Perucca E Ryvlin P Tomson T. Epilepsy: new advances. Lancet. (2015) 385:884-98. 10.1016/S0140-6736(14)60456-6 25260236

[2] Schomer AC Nearing BD Schachter SC Verrier RL. Vagus nerve stimulation reduces cardiac electrical instability assessed by quantitative T-wave alternans analysis in patients with drug-resistant focal epilepsy. Epilepsia. (2014) 55:1996–2002. 10.1111/epi.12855 25470430

[3] Kwan P Arzimanoglou A Berg AT Brodie MJ Allen Hauser W Mathern G. Definition of drug resistant epilepsy: consensus proposal by the ad hoc task force of the ILAE commission on therapeutic strategies. Epilepsia. (2010) 51:1069-77. 10.1111/j.1528-1167.2009.02397.x 19889013

[4] Yang J Ji HP. The present and future of vagus nerve stimulation. J Korean Neurosurg Soc. (2019) 62:344-52. 10.3340/jkns.2019.0037 31085961 PMC6514309

[5] Englot DJ Chang EF Auguste KI. Vagus nerve stimulation for epilepsy: a meta-analysis of efficacy and predictors of response: a review. J Neurosurg. (2011) 115:1248-55. 10.3171/2011.7.JNS11977 21838505

[6] Mogul DJ Van DW. Electrical control of epilepsy. Ann Rev Biomed Eng. (2014) 16:483-504. 10.1146/annurev-bioeng-071813-104720 25014790

[7] Liu H Yang Z Huang L Qu W Hao H Li L. Heart-rate variability indices as predictors of the response to vagus nerve stimulation in patients with drug-resistant epilepsy. Epilepsia. (2017) 58:1015-22. 10.1111/epi.13738 28440954

[8] Liu HY Yang Z Meng FG Guan YG Ma YS Liang SL. Preoperative heart rate variability as predictors of vagus nerve stimulation outcome in patients with drug-resistant epilepsy. Sci Rep. (2018) 8:1-11. 10.1038/s41598-018-21669-3 29497072 PMC5832772

[9] Hödl S Carrette S Meurs A Carrette E Mertens A Gadeyne S. Neurophysiological investigations of drug resistant epilepsy patients treated with vagus nerve stimulation to differentiate responders from non-responders. Eur J Neurol. (2020) 27:1178-89. 10.1111/ene.14270 32310326

[10] Cumming G. The new statistics: why and how. Psychol Sci. (2014) 25:7-29. 10.1177/0956797613504966 24220629

[11] Durlak JA. How to select, calculate, and interpret effect sizes. J Pediatr Psychol. (2009) 34:917-28. 10.1093/jpepsy/jsp004 19223279

[12] Vasilopoulos T Morey TE Dhatariya K Rice MJ. Limitations of significance testing in clinical research: a review of multiple comparison corrections and effect size calculations with correlated measures. Anesth Analg. (2016) 122:825-30. 10.1213/ANE.0000000000001107 26891394

[13] Wuest T Weimer D Irgens C Thoben KD. Machine learning in manufacturing: advantages, challenges, and applications. Prod Manuf Res. (2016) 4:23-45. 10.1080/21693277.2016.1192517

[14] Li J Tian Y Zhu Y Zhou T Li J Ding K. A multicenter random forest model for effective prognosis prediction in collaborative clinical research network. Artif Intell Med. (2020) 103:101814. 10.1016/j.artmed.2020.101814 32143809

[15] Ibrahim GM Sharma P Hyslop A Guillen MR Morgan BR Wong S. Presurgical thalamocortical connectivity is associated with response to vagus nerve stimulation in children with intractable epilepsy. Neuroimage Clin. (2017) 16:634-42. 10.1016/j.nicl.2017.09.015 28971013 PMC5619991

[16] Mithani K Mikhail M Morgan BR Wong S Weil AG Deschenes S. Connectomic profiling identifies responders to vagus nerve stimulation. Ann Neurol. (2019) 86:743-53. 10.1002/ana.25574 31393626

[17] Mithani K Wong SM Mikhail M Pourmotabbed H Pang E Sharma R. Somatosensory evoked fields predict response to vagus nerve stimulation. Neuroimage Clin. (2020) 26:102205. 10.1016/j.nicl.2020.102205 32070812 PMC7026289

[18] Massin MM Maeyns K Withofs N Ravet F Gérard P. Circadian rhythm of heart rate and heart rate variability. Arch Dis Child. (2000) 83:179-82. 10.1136/adc.83.2.179 10906034 PMC1718415

[19] Philippe B Wei-Hsien Y Dumont GA Boivin DB. Circadian variation of heart rate variability across sleep stages. Sleep. (2013) 12:1919. 10.5665/sleep.3230 24293767 PMC3825442

[20] Manfredini R Boari B Bressan S Gallerani M Salmi R Portaluppi F. Influence of circadian rhythm on mortality after myocardial infarction: data from a prospective cohort of emergency calls. Am J Emerg Med. (2004) 22:555-9. 10.1016/j.ajem.2004.08.014 15666260

[21] Ronkainen E Ansakorpi H Huikuri HV Myllylä VV Isojärvi JIT Korpelainen JT. Suppressed circadian heart rate dynamics in temporal lobe epilepsy. J Neurol Neurosurg Psychiatry. (2005) 76:1382-6. 10.1136/jnnp.2004.053777 16170081 PMC1739357

[22] Balasubramanian K Harikumar K Nagaraj N Pati S. Vagus nerve stimulation modulates complexity of heart rate variability differently during sleep and wakefulness. Ann Indian Acad Neurol. (2017) 20:403. 10.4103/aian.AIAN_148_17 29184345 PMC5682746

[23] Balasubramanian K Nagaraj N Pati S. Chaos or randomness? Effect of vagus nerve stimulation during sleep on heart-rate variability. IETE J Res. (2020) 11:1–7. 10.1080/03772063.2020.178016531358702

[24] Lin YH Wu VC Lo MT Wu XM Hung CS Wu KD. Reversible heart rhythm complexity impairment in patients with primary aldosteronism. Sci Rep. (2015) 5:1-10. 10.1038/srep11249 26282603 PMC4539539

[25] Lin YH Lin C Ho YH Wu VC Lo MT Hung KY. Heart rhythm complexity impairment in patients undergoing peritoneal dialysis. Sci Rep. (2016) 6:1-19. 10.1038/srep28202 27324066 PMC4914979

[26] Makowski D Pham T Lau ZJ Brammer JC Lespinasse F Pham H. NeuroKit2: a Python toolbox for neurophysiological signal processing. Behav Res Methods. (2021) 51:1–8. 10.3758/s13428-020-01516-y 33528817

[27] Heart rate variability: standards of measurement, physiological interpretation and clinical use. Task Force of the European Society of Cardiology and the North American Society of Pacing and Electrophysiology. Circulation. (1996) 93:1043–65. 10.1111/j.1542-474X.1996.tb00275.x 8598068

[28] Stein PK. Assessing heart rate variability from real-world Holter reports. Card Electrophysiol Rev. (2002) 6:239-44. 10.1023/A:1016376924850 12114845

[29] Shaffer F Ginsberg JP. An overview of heart rate variability metrics and norms. Front Public Health. (2017) 5:258. 10.3389/fpubh.2017.00258 29034226 PMC5624990

[30] Bachler M. Spectral analysis of unevenly spaced data: models and application in heart rate variability. Simul Notes Eur. (2017) 27:183-90. 10.11128/sne.27.tn.10393

[31] Costa MD Davis RB Goldberger AL. Heart rate fragmentation: a new approach to the analysis of cardiac interbeat interval dynamics. Front Physiol. (2017) 8:255. 10.3389/fphys.2017.00255 28536533 PMC5422439

[32] Yan C Li P Ji L Yao L Karmakar C Liu C. Area asymmetry of heart rate variability signal. Biomed Eng Online. (2017) 16:112. 10.1186/s12938-017-0402-3 28934961 PMC5607847

[33] Piskorski J Guzik P. Asymmetric properties of long-term and total heart rate variability. Med Biol Eng Comput. (2011) 49:1289-97. 10.1007/s11517-011-0834-z 21953298 PMC3208812

[34] Shaffer F McCraty R Zerr CL. A healthy heart is not a metronome: an integrative review of the heart's anatomy and heart rate variability. Front Psychol. (2014) 5:1040. 10.3389/fpsyg.2014.01040 25324790 PMC4179748

[35] Billman GE Huikuri HV Sacha J Trimmel K. An introduction to heart rate variability: methodological considerations and clinical applications. Front Physiol. (2015) 6:55. 10.3389/fphys.2015.00055 25762937 PMC4340167

[36] Brennan M Palaniswami M Kamen P. Do existing measures of Poincare plot geometry reflect nonlinear features of heart rate variability? IEEE Trans Biomed Eng. (2001) 48:1342-7. 10.1109/10.959330 11686633

[37] Saeys Y Inza I Larranaga P. A review of feature selection techniques in bioinformatics. Bioinformatics. (2007) 23:2507-17. 10.1093/bioinformatics/btm344 17720704

[38] Yu L Liu H. Feature selection for high-dimensional data: a fast correlation-based filter solution. In: Proceedings of The Twentieth International Conference on Machine Leaning (ICML-03). Washington DC (2003) p. 856-63.

[39] Varma S Simon R. Bias in error estimation when using cross-validation for model selection. BMC Bioinformatics. (2006) 7:91. 10.1186/1471-2105-7-91 16504092 PMC1397873

[40] Varoquaux G Raamana PR Engemann D Hoyos-Idrobo A Schwartz Y Thirion B. Assessing and tuning brain decoders: cross-validation, caveats, and guidelines. Neuroimage. (2017) 145:166-79. 10.1016/j.neuroimage.2016.10.038 27989847

[41] Bolón-Canedo V Alonso-Betanzos A. Ensembles for feature selection: a review and future trends. Inf Fusion. (2019) 52:1-12. 10.1016/j.inffus.2018.11.008

[42] Portaluppi F Tiseo R Smolensky MH Hermida RC Ayala DE Fabbian F. Circadian rhythms and cardiovascular health. Sleep Med Rev. (2012) 16:151-66. 10.1016/j.smrv.2011.04.003 21641838

[43] Guo YF Stein PK. Circadian rhythm in the cardiovascular system: considerations in non-invasive electrophysiology. Card Electrophysiol Rev. (2002) 6:267-72. 10.1023/A:1016337210738 12114850

[44] Bilan A Witczak A Palusiński R Myśliński W Hanzlik J. Circadian rhythm of spectral indices of heart rate variability in healthy subjects. J Electrocardiol. (2005) 38:239-43. 10.1016/j.jelectrocard.2005.01.012 16003709

[45] Boudreau P Yeh WH Dumont GA Boivin DB. A circadian rhythm in heart rate variability contributes to the increased cardiac sympathovagal response to awakening in the morning. Chronobiol Int. (2012) 29:757-68. 10.3109/07420528.2012.674592 22734576

[46] Hodl S Carrette E Meurs A Dewaele F Carrette S Raedt R. VNS responders have a significant higher amount of deep sleep-The Adenosine Hypothesis. Eur J Neurol. (2019) 26:102. Available online at: http://hdl.handle.net/1854/LU-8703832

[47] Chavel SM Westerveld M Spencer S. Long-term outcome of vagus nerve stimulation for refractory partial epilepsy. Epilepsy Behav. (2003) 4:302–9. 10.1016/S1525-5050(03)00109-4 12791333

[48] Lotufo PA Valiengo L Bensen IM Brunoni AR. A systematic review and meta-analysis of heart rate variability in epilepsy and antiepileptic drugs. Epilepsia. (2012) 53:272–82. 10.1111/j.1528-1167.2011.03361.x 22221253

[49] Beckers F Ramaekers D Aubert AE. Approximate entropy of heart rate variability: validation of methods and application in heart failure. Cardiovasc Eng. (2001) 1:177–82. 10.1023/A:1015212328405

[50] Lippman NEAL Stein KM Lerman BB. Comparison of methods for removal of ectopy in measurement of heart rate variability. Am J Physiol. (1994) 267:411-8. 10.1152/ajpheart.1994.267.1.H411 7519408

[51] Brodesser D Frick C Hosten S Axmacher N Schlaepfer TE. P.2.a.025 predictors of response to vagus nerve stimulation. Eur Neuropsychopharmacol. (2006) 16:S296-7. 10.1016/S0924-977X(06)70305-4

[52] Janszky J Hoppe M Behne F Tuxhorn I Pannek HW Ebner A. Vagus nerve stimulation: predictors of seizure freedom. J Neurol Neurosurg Psychiatry. (2005) 76:384-9. 10.1136/jnnp.2004.037085 15716532 PMC1739542

[53] Englot DJ Rolston JD Wright CW Hassnain KH Chang EF. Rates and predictors of seizure freedom with vagus nerve stimulation for intractable epilepsy. Neurosurgery. (2016) 79:345-53. 10.1227/NEU.0000000000001165 26645965 PMC4884552

[54] Xiong J Cao Y Yang W Chen Z Yu Q. Can we predict response to vagus nerve stimulation in intractable epilepsy. Int J Neurosci. (2020) 130:1063-70. 10.1080/00207454.2020.1713777 31914344

[55] Brázdil M DoleŽalová I Koritáková E Chládek J Roman R Pail M. EEG reactivity predicts individual efficacy of vagal nerve stimulation in intractable epileptics. Front Neurol. (2019) 10:392. 10.3389/fneur.2019.00392 31118916 PMC6507513

[56] Meisel C Loddenkemper T. Seizure prediction and intervention. Neuropharmacology. (2020) 172:107898. 10.1016/j.neuropharm.2019.107898 31839204

[57] Ufongene C El Atrache R Loddenkemper T Meisel C. Electrocardiographic changes associated with epilepsy beyond heart rate and their utilization in future seizure detection and forecasting methods. Clin Neurophysiol. (2020) 131866-79. 10.1016/j.clinph.2020.01.007 32066106

