# Supplementary material for: Preoperative Heart Rate Variability During Sleep Predicts Vagus Nerve Stimulation Outcome Better in Patients With Drug-Resistant Epilepsy
Source: Front Neurol. 2021 Jul 7;12:691328. doi: 10.3389/fneur.2021.691328 (PMC8292667; doi:10.3389/fneur.2021.691328)
Supplement: Supplementary file 1 [file Data_Sheet_1.PDF]

Supplementary Material

Demographic data	DRE patients (n=59)	Healthy controls (n=50)	p value
Age(years)	19.2±8.3	21.0±7.8	0.181
Male/female	40/19	34/16	1.000
BMI(kg/m^2)	22.3±4.3	21.7±3.3	0.225

Table S1. Demographic data of all study population. Data were presented as mean value ± standard deviation. DRE, drug-resistant epilepsy.

HRV indices	Sleep			Awake		
	DRE patients (n=59)	Healthy controls (n=50)	p Value	DRE patients (n=59)	Healthy controls (n=50)	p Value
RMSSD	45.5±23.8	61.4±28.2	0.002	24.5±12.5	31.7±13.4	0.001
MeanNN	857.5±136.0	952.3±131.0	<0.001	644.7±91.3	694.1±113.7	0.019
SDNN	78.9±25.2	102.4±29.2	<0.001	68.8±22.5	85.9±25.3	<0.001
SDSD	45.5±23.8	61.4±28.2	0.002	24.5±12.5	31.7±13.4	0.001
CVNN	0.09±0.02	0.00±0.00	<0.001	0.11±0.03	0.00±0.00	<0.001
CVSD	0.05±0.03	0.00±0.00	<0.001	0.04±0.02	0.00±0.00	<0.001
MedianNN	863.1±141.6	962.1±137.7	<0.001	643.6±92.5	691.4±117.1	0.022
MadNN	67.3±26.1	85.6±28.5	<0.001	69.7±27.9	85.1±28.9	0.002
MCVNN	0.08±0.03	0.00±0.00	<0.001	0.11±0.03	0.00±0.00	<0.001
IQRNN	92.2±37.3	119.0±42.6	<0.001	95.7±38.5	118.7±41.6	0.001
pNN50	23.1±17.4	34.7±19.4	0.002	6.0±7.8	11.0±9.6	0.001
pNN20	53.8±19.7	64.7±16.5	0.002	25.5±16.8	37.1±17.2	<0.001
TINN	657.9±172.3	788.9±195.9	<0.001	578.8±143.6	639.0±139.9	0.017
HTI	20.7±1.8	20.0±2.1	0.039	22.2±2.3	21.9±2.2	0.037
ULF	68.4±192.0	10.9±47.5	0.105	459.1±483.8	407.8±442.6	0.428
VLF	1698.7±1325.7	2884.6± 1741.4	<0.001	1159.7±854.1	1988.5±1143.4	<0.001
LF	1344.7±1084.5	2581.4± 1493.4	<0.001	638.1±568.0	1122.3±770.9	<0.001
HF	678.3±584.1	1146.8±740.5	<0.001	409.3±365.3	787.0±437.4	<0.001
VHF	90.9±76.5	251.4±443.6	<0.001	68.5±86.8	153.5±142.6	<0.001
LF/HF	2.15±0.82	1.90±0.70	0.189	1.79±0.64	1.98±0.55	0.377
LFn	0.33±0.08	0.00±0.00	<0.001	0.22±0.06	0.00±0.00	<0.001
HFn	0.17±0.05	0.00±0.00	<0.001	0.14±0.06	0.00±0.00	<0.001

LnHF	6.21±0.86	6.56±0.82	0.004	5.66±0.85	5.98±0.76	0.007
SD1	32.2±16.8	43.2±19.9	0.003	17.3±8.9	22.3±9.5	0.001
SD2	106.3±33.5	137.8±38.2	<0.001	95.4±31.3	119.4±35.1	<0.001
SD1/SD2	0.30±0.11	0.00±0.00	<0.001	0.18±0.07	0.00±0.00	<0.001
S	11969.0± 10141.6	20768.5± 13378.8	<0.001	5704.2±4341.8	9399.2±6247.2	<0.001
CSI	3.88±1.57	3.02±1.19	0.002	6.23±2.15	5.36±2.08	0.014
CVI	4.66±0.35	4.26±0.54	<0.001	4.35±0.31	4.04±0.40	<0.001
CSI_ Modified	1606.6±696.0	1918.3±696.4	0.027	2399.5±1291.1	2717.1±971.7	0.030
PIP	0.51±0.05	0.00±0.00	<0.001	0.50±0.06	0.00±0.00	<0.001
IALS	0.50±0.05	0.00±0.00	<0.001	0.49±0.06	0.00±0.00	<0.001
PSS	0.75±0.10	0.00±0.00	<0.001	0.73±0.08	0.00±0.00	<0.001
PAS	0.06±0.04	0.00±0.00	<0.001	0.09±0.05	0.00±0.00	<0.001
GI	50.0±0.0	49.8±0.4	0.005	50.0±0.0	49.7±0.5	<0.001
SI	50.0±0.0	49.4±0.5	<0.001	50.0±0.0	49.5±0.5	<0.001
AI	50.0±0.0	49.9±0.3	<0.001	50.0±0.0	49.8±0.4	<0.001
PI	50.6±2.8	50.9±2.5	0.788	50.2±2.3	49.4±2.8	0.157
C1d	0.52±0.04	0.00±0.00	<0.001	0.51±0.03	0.00±0.00	<0.001
C1a	0.48±0.04	0.00±0.00	<0.001	0.49±0.03	0.00±0.00	<0.001
SD1d	23.2±12.4	31.6±15.0	0.002	12.4±6.5	15.7±6.8	0.002
SD1a	22.3±11.4	29.2±13.1	0.005	12.1±6.1	15.4±6.8	0.003
C2d	0.46±0.03	0.00±0.00	<0.001	0.49±0.02	0.00±0.00	<0.001
C2a	0.54±0.03	0.00±0.00	<0.001	0.51±0.02	0.00±0.00	<0.001
SD2d	71.7±21.6	90.8±24.2	<0.001	67.0±21.7	83.9±24.1	<0.001
SD2a	78.4±25.8	103.2±29.7	<0.001	67.9±22.7	84.8±25.6	<0.001
Cd	0.46±0.03	0.00±0.00	<0.001	0.49±0.02	0.00±0.00	<0.001
Ca	0.54±0.03	0.00±0.00	<0.001	0.51±0.02	0.00±0.00	<0.001
SDNNd	53.6±16.6	68.3±19.0	<0.001	48.3±15.6	60.3±17.6	<0.001
SDNNa	57.9±19.1	76.0±22.1	<0.001	48.9±16.3	60.9±18.5	<0.001
ApEn	1.39±0.25	1.00±0.00	<0.001	0.98±0.28	0.64±0.48	0.002
SampEn	1.27±0.25	0.94±0.24	<0.001	0.80±0.26	0.30±0.46	<0.001

Table S2. A total of 52 preoperative HRV indices from NeuroKit2 of DRE patients and healthy controls. Data were presented as mean value ± standard deviation. DRE, drug-resistant epilepsy.

HRV indices	Sleep		Awake		p1 value	p2 value	p3 value	p4 value
	Responders	Non-responders	Responders	Non-responders				
RMSSD	54.5±26.2	34.9±15.0	27.0±13.1	21.6±9.8	<0.001	0.025	<0.001	<0.001
MeanNN	896.4±124.6	835.5±138.3	673.3±85.6	630.5±87.4	0.031	0.041	<0.001	<0.001
SDNN	87.0±26.3	70.7±21.6	70.6±21.6	68.4±24.0	0.005	0.306	0.004	0.619
SDSD	54.5±26.2	34.9±15.0	27.0±13.1	21.6±9.8	<0.001	0.025	<0.001	<0.001
CVNN	0.10±0.02	0.08±0.02	0.10±0.02	0.11±0.03	0.008	0.395	0.311	0.002
CVSD	0.06±0.03	0.04±0.02	0.04±0.02	0.03±0.01	0.001	0.052	<0.001	0.075
MedianNN	902.2±130.8	842.0±143.5	672.9±86.4	629.1±88.6	0.034	0.036	<0.001	<0.001
MadNN	76.7±29.9	56.5±14.7	69.9±25.9	71.7±30.7	<0.001	0.497	0.112	0.096
MCVNN	0.08±0.03	0.07±0.01	0.10±0.03	0.11±0.04	0.002	0.204	0.002	<0.001
IQRNN	105.4±43.4	77.0±20.4	96.2±35.2	98.2±43.0	<0.001	0.497	0.158	0.114
pNN50	30.2±17.3	14.9±13.4	7.94±8.79	3.75±5.48	0.001	0.014	<0.001	<0.001
pNN20	60.1±19.1	46.8±18.2	31.3±17.3	19.8±13.7	0.002	0.006	<0.001	<0.001
TINN	690.9±160.7	631.6±185.9	577.8±115.5	587.0±169.3	0.039	0.431	0.003	0.213
HTI	20.4±1.85	20.8±1.7	22.2±2.3	21.9±2.4	0.213	0.413	<0.001	0.018
ULF	171.2±209.3	240.0±248.6	318.3±255.0	241.8±185.8	0.083	0.09	0.002	0.464
VLF	2079.9±1566.0	1423.0±958.1	1338.4±848.5	1020.0±866.0	0.019	0.026	0.05	0.052
LF	1569.3±1214.9	1097.3±759.9	715.6±496.4	593.5±653.6	0.037	0.047	<0.001	0.001
HF	832.4±717.4	525.4±360.3	490.8±369.5	357.5±361.8	0.010	0.041	0.004	0.031
VHF	119.6±83.8	62.1±53.7	76.2±63.5	66.6±108.6	0.001	0.083	0.014	0.32
LF/HF	2.12±0.92	2.22±0.72	1.74±0.73	1.77±0.51	0.226	0.183	0.283	0.016
LFn	0.33±0.07	0.33±0.08	0.23±0.06	0.22±0.06	0.425	0.316	<0.001	<0.001
HFn	0.18±0.06	0.16±0.05	0.15±0.06	0.14±0.05	0.209	0.222	0.01	0.083

LnHF	6.42±0.82	5.97±0.86	5.88±0.85	5.51±0.83	0.010	0.041	0.004	0.03
SD1	38.5±18.5	24.7±10.6	19.1±9.2	15.3±7.0	<0.001	0.025	<0.001	<0.001
SD2	116.1±34.7	96.5±29.7	97.7±29.9	95.4±33.6	0.011	0.285	0.012	0.744
SD1/SD2	0.33±0.12	0.26±0.09	0.20±0.07	0.16±0.04	0.005	0.052	<0.001	<0.001
S	15382.1±12014.7	8133.5±5719.7	6383.8±4516.9	5080.9±4029.3	<0.001	0.105	<0.001	0.006
CSI	3.59±1.70	4.29±1.35	5.86±2.17	6.67±2.02	0.005	0.052	<0.001	<0.001
CVI	4.78±0.34	4.52±0.30	4.41±0.31	4.32±0.30	<0.001	0.105	<0.001	0.008
CSI_Modified	1606.8±727.3	1660.5±675.1	2271.9±1008.2	2625.2±1543.4	0.355	0.285	0.009	0.007
PIP	0.52±0.05	0.5±0.05	0.51±0.06	0.49±0.05	0.076	0.092	0.83	0.517
IALS	0.51±0.05	0.49±0.05	0.49±0.06	0.47±0.05	0.041	0.076	0.472	0.177
PSS	0.78±0.11	0.73±0.1	0.74±0.08	0.71±0.08	0.028	0.087	0.254	0.346
PAS	0.06±0.04	0.06±0.04	0.09±0.04	0.09±0.05	0.222	0.431	<0.001	0.007
GI	50.0±0.01	50.0±0.01	50.01±0.02	50.01±0.02	0.08	0.425	0.084	0.433
SI	49.99±0.02	50.0±0.01	50.0±0.02	50.0±0.03	0.003	0.290	0.125	0.524
AI	50.0±0.0	50.0±0.0	50.0±0.0	50.0±0.0	0.395	0.231	0.452	0.092
PI	51.0±2.6	50.1±2.9	49.9±2.4	50.3±2.0	0.076	0.333	0.153	0.913
C1d	0.52±0.03	0.51±0.04	0.51±0.04	0.52±0.03	0.143	0.231	0.257	0.748
C1a	0.48±0.03	0.49±0.04	0.49±0.04	0.48±0.03	0.143	0.231	0.254	0.772
SD1d	27.9±13.7	17.7±7.6	13.6±6.6	11.0±5.2	<0.001	0.030	<0.001	<0.001
SD1a	26.5±12.5	17.2±7.4	13.4±6.5	10.6±4.6	0.001	0.026	<0.001	<0.001
C2d	0.46±0.03	0.46±0.03	0.50±0.02	0.49±0.02	0.25	0.473	<0.001	<0.001
C2a	0.54±0.03	0.54±0.03	0.50±0.02	0.51±0.02	0.25	0.473	<0.001	<0.001
SD2d	77.9±21.7	65.2±19.9	68.6±20.6	67.0±23.4	0.009	0.344	0.029	0.938
SD2a	86.0±27.2	71.1±22.3	69.6±21.9	67.9±24.2	0.010	0.306	0.005	0.524
Cd	0.46±0.02	0.46±0.03	0.50±0.02	0.49±0.02	0.322	0.479	<0.001	<0.001
Ca	0.54±0.02	0.54±0.03	0.5±0.02	0.51±0.02	0.322	0.479	<0.001	<0.001
SDNNd	58.9±16.9	47.9±14.5	49.6±14.9	48.1±16.7	0.004	0.311	0.009	0.938
SDNNa	64.0±20.2	51.87±16.1	50.2±15.8	48.6±17.3	0.005	0.295	0.002	0.455

ApEn	1.44±0.27	1.32±0.20	1.07±0.3	0.89±0.22	0.007	0.010	<0.001	<0.001
SampEn	1.31±0.27	1.20±0.20	0.89±0.28	0.71±0.21	0.008	0.009	<0.001	<0.001

Table S3. A total of 52 preoperative HRV indices from NeuroKit2 of responders and non-responders. Data were presented as mean value ± standard deviation. P-value were provided for comparison as follows: (1) p1: responders vs. non-responders in sleep state; (2) p2: responders vs. non-responders in awake state; (3) p3: sleep vs. awake state of responders; (4) p4: sleep vs. awake state of non-responders.

**Filter Methods**		**Sleep**				**Awake**			
		Acc./%	*Pre./%*	*Rec./%*	*F1/%*	*Acc./%*	*Pre./%*	*Rec./%*	*F1/%*
Different relevance measurements	Chi2	74.6	80.0	70.6	75.0	70.7	78.5	72.8	70.3
	Anova F-value	71.4	76.7	73.5	71.2	66.7	68.6	70.6	69.6
	MI	69.8	72.7	73.5	70.4	65.1	65.7	76.5	70.3
Multivariate filter (FCBF)		71.0	74.5	77.5	73.0	57.4	59.8	73.3	57.3

Table S4. Prediction performances of other univariate filter measurements and multivariate filter compared to chi-squared statistics. Chi2, Chi-squared statistics; MI, mutual information; FCBF, fast correlation-based filter.

States	Classifier	5-fold Cross-validation				LOO			
		Acc./%	*Pre./%*	*Rec./%*	*F1/%*	*Acc./%*	*Pre./%*	*Rec./%*	*F1/%*
**RFE_sleep**	RF	71.4	77.4	71.0	70.5	73.4	80.3	86.4	77.9
	SVM	68.1	86.8	88.6	72.8	65.5	68.4	69.2	64.3
	LDA	69.7	75.2	73.8	71.5	69.3	73.7	70.8	69.1
**RFE_awake**	RF	68.7	70.7	67.6	65.5	64.9	74.0	70.3	65.7
	SVM	65.0	89.3	88.6	68.9	66.7	67.5	77.5	59.7
	LDA	68.5	71.7	73.8	67.1	63.3	71.7	72.5	67.0
**UF_sleep**	RF	78.3	73.6	71.4	70.0	74.6	80.0	70.6	75.0
	SVM	65.8	58.3	80.0	66.4	63.5	69.0	61.8	63.5
	LDA	68.7	63.0	62.4	61.5	66.7	71.0	67.6	68.7
	KNN	68.1	71.0	70.5	70.5	63.5	65.7	73.5	68.5
	GNB	61.9	74.1	50.0	58.4	68.3	81.8	52.9	64.3
**UF_awake**	RF	66.8	74.2	73.3	69.1	65.3	66.4	70.5	68.4
	SVM	58.8	71.5	94.3	70.4	62.0	65.6	70.6	64.9
	LDA	61.0	65.5	61.4	62.5	64.5	68.8	70.6	69.6
	KNN	58.5	59.8	67.6	63.2	58.7	61.1	64.7	62.9
	GNB	58.5	65.7	46.2	52.5	61.9	77.8	50.0	56.7

Table S5. Comparison of prediction performances of different classifiers and 5-fold cross-validation. RF, Random Forest; SVM, Chi-squared statistics; LDA, Linear Discriminant Analysis; KNN, K-Nearest Neighbors; GNB, Gaussian Naïve Bayes; LOO, Leave One Out.

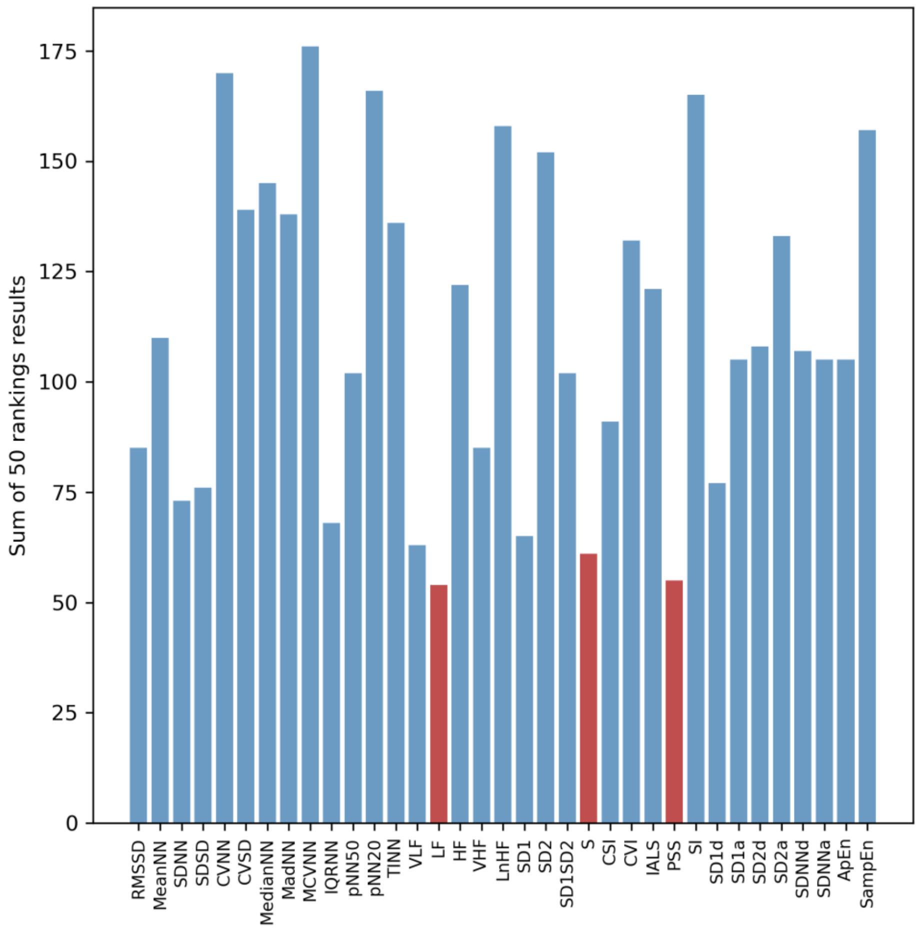

Figure S1. Accumulated ranking results of each HRV index in sleep state through 50 times computation of RFE algorithm. The top three indices of ranking are shown in red bars (LF, PSS, S).

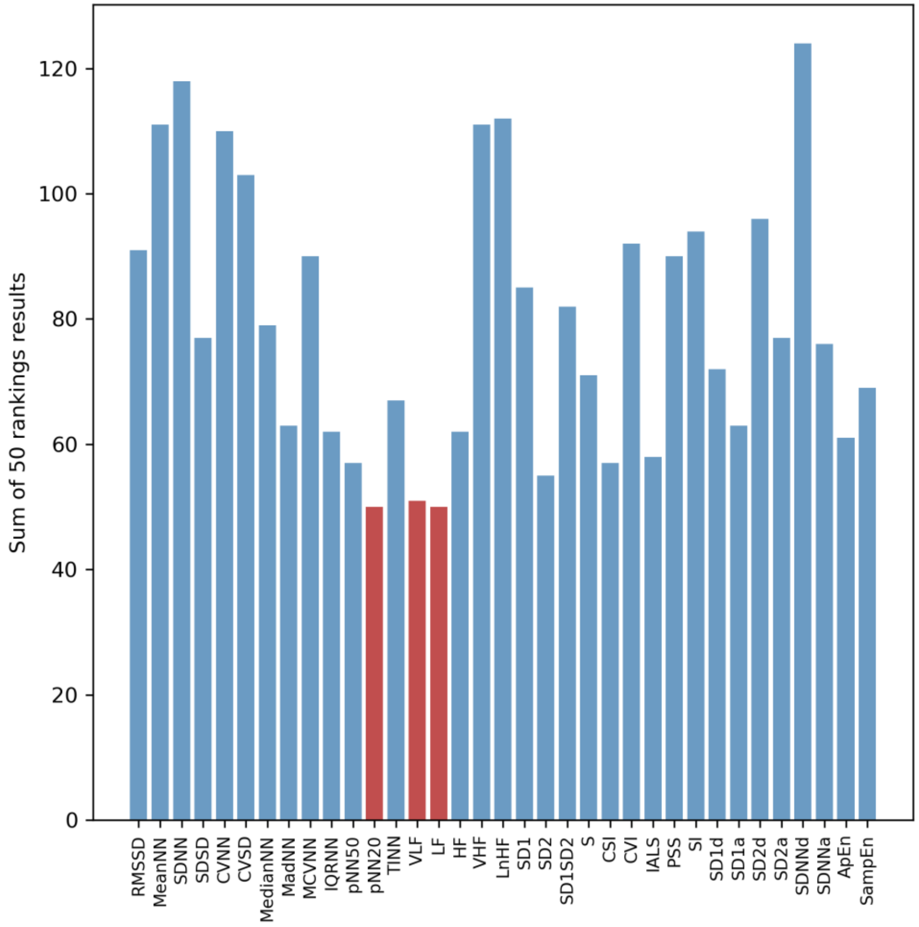

Figure S2. Accumulated ranking results of each HRV index in awake state through 50 times computation of RFE algorithm. The top three indices of ranking are shown in red bars (LF, pNN20, VLF).
